# Endoscopic transgastric necrosectomy for infected necrotizing pancreatitis extending from the lesser Sac to the paracolic gutter in a pediatric patient: a case report

**DOI:** 10.3389/fmed.2026.1958448

**Published:** 2026-09-16

**Authors:** Ximei Cao, Yao Xu, Nonghua Lv, Yin Zhu, Wenhua He

**Affiliations:** 1 Department of Gastroenterology, Jiujiang City Key Laboratory of Cell Therapy, Jiujiang No.1 People's Hospital, Jiujiang, Jiangxi, China; 2 Department of Gastroenterology, Digestive Disease Hospital, The First Affiliated Hospital of Nanchang University, Nanchang, Jiangxi, China

**Keywords:** endoscopic transgastric necrosectomy, infected necrotizing pancreatitis, paracolic gutter, pediatric, walled-off necrosis

## Abstract

Endoscopic transgastric necrosectomy (ETN) has emerged as a preferred minimally invasive approach for managing walled-off necrosis (WON); however, its conventional indication remains confined to necrosis limited to the lesser sac. The applicability of this technique for extensive necrosis extending to the paracolic gutter and pelvic cavity remains poorly characterized, particularly in pediatric patients. We herein report the case of a 7-year-old boy with infected necrotizing pancreatitis, in whom imaging demonstrated necrosis extending from the lesser sac to the bilateral paracolic gutters, iliac fossae, and pelvic cavity. Following initial conservative management and CT-guided percutaneous drainage, endoscopic ultrasonography (EUS)-guided transmural drainage was performed at week 4, followed by ETN on day 40. During the procedure, the conventional indication was extended by leveraging the flexibility of the endoscope to navigate from the pancreatic tail into the left paracolic gutter and pelvic cavity, thereby achieving successful debridement without the need for additional percutaneous sinus endoscopy or video-assisted retroperitoneal debridement. The patient recovered uneventfully, with post-procedural CT confirming complete resolution and stents removed three months later. This case suggests that extended ETN may represent a safe and effective option for managing extensive WON beyond the lesser sac in children, potentially obviating the need for more invasive combined procedures.

## Introduction

The incidence of acute pancreatitis (AP) in children has been steadily increasing. A study of pediatric AP cases in the United States between 2007 and 2014 reported an incidence ranging from 3.6 to 13.2 per 100,000 children, whereas the estimated annual incidence in adults is approximately 40 per 100,000 (1). A recent systematic review and meta-analysis of the global incidence of AP, encompassing studies published over a 56-year period (1961–2016), found that the overall global incidence of AP increased by 3.07% per year, with a pooled annual increase of 5.44% in the pediatric population (2). The INSPPIRE (INternational Study Group of Pediatric Pancreatitis: In Search for a CuRE) definition of pediatric AP is an expert consensus definition modeled after the Atlanta criteria in adults (3). Although the majority of children with AP experience mild disease, a substantial proportion require admission to the intensive care unit for transient multi-organ dysfunction (4); fortunately, overall mortality remains below 5% (5).

Most cases of AP resolve with conservative medical management. However, approximately 20% of patients develop necrotizing pancreatitis, which is strongly associated with complications and high mortality (6). Pancreatic fluid collections (PFCs) are a common complication of AP, typically arising during the acute phase and often resolving spontaneously. The revised Atlanta classification stratifies PFCs based on duration, the presence of a capsule, and contents into acute peripancreatic fluid collection (APFC), acute necrotic collection (ANC), pancreatic pseudocyst (PPC), and walled-off necrosis (WON) (7). WON is a mature, encapsulated cystic-solid structure containing pancreatic and/or peripancreatic necrotic tissue. Over the past decade, the management of WON has progressively shifted from traditional open surgery toward minimally invasive surgical and endoscopic interventions. With the well-established clinical application of endoscopic ultrasonography (EUS) and the concurrent development of related techniques and accessories, EUS-guided stent drainage has become the first-line treatment for WON (8). A multicenter randomized trial demonstrated that clinical success could be achieved with transmural drainage and placement of either plastic or metal stents in 41% of WON patients, without the need for additional necrosectomy (9). When stent drainage fails to improve clinical symptoms or when the collection contains a substantial amount of solid necrotic debris, endoscopic necrosectomy (EN) is indicated (10). However, experience with EN in pediatric patients with extensive necrosis extending to the paracolic gutter and pelvic cavity remains extremely limited. Herein, we report the case of a child in whom extended endoscopic transgastric necrosectomy (ETN) was performed to achieve successful debridement of extensive WON extending to the paracolic gutter.

## Case description

A 7-year-old boy presented with a 3-day history of abdominal pain following excessive food intake, accompanied by vomiting. His past medical history was notable for an episode of acute pancreatitis one year prior. On admission, his vital signs were as follows: temperature 37.1 °C, heart rate 128 beats/min, respiratory rate 22 breaths/min, blood pressure 128/81 mmHg, body weight 30 kg. Laboratory investigations revealed serum amylase of 852.0 U/L, lipase of 1,290.0 U/L, white blood cell count 27.07 × 10⁹/L, and C-reactive protein 68.70 mg/L. The serum triglyceride level measured at the referring hospital was 60 mmol/L, establishing the diagnosis of hypertriglyceridemia-induced acute pancreatitis. MRCP showed no dilation, stricture, or filling defect of the main pancreatic duct; abdominal ultrasound and MRCP excluded biliary disease and pancreatic anatomical abnormalities; genetic testing revealed no hereditary pancreatitis-related mutations; autoimmune markers and medication history were negative, excluding other potential etiologies.

On day 2 of illness, circulatory failure (hypovolemic shock) developed, which resolved after 2 days of fluid resuscitation without the need for vasoactive agents; oxygenation index was >300 mmHg with no respiratory failure or renal dysfunction. The child was transferred to our pancreatic ICU on day 4 and stayed for 18 days, meeting the criteria for “severe acute pancreatitis” according to the Revised Atlanta Classification. CT at one week showed pancreatic and peripancreatic necrosis extending to the bilateral paracolic gutters, iliac fossae, and pelvic cavity (Figure 1). Persistent high fever developed from day 8 onward. CT-guided percutaneous drainage was performed during weeks 2–3 (Figures 2a,b), and bacterial culture of the drainage fluid yielded Klebsiella pneumoniae and Enterococcus faecium, confirming infected necrotizing pancreatitis. At week 4, EUS revealed a 5.3 cm × 6.8 cm collection in the pancreatic body and tail with necrotic debris comprising less than 20% of the cavity volume. Two double-pigtail plastic stents (7 Fr × 5.0 cm and 10 Fr × 3.0 cm) were placed via a high posterior gastric body puncture (Figures 2c,d). Post-procedural CT still showed extensive necrotic tissue in the peripancreatic region and paracolic gutter (Figures 2e,f).

**Figure 1:**
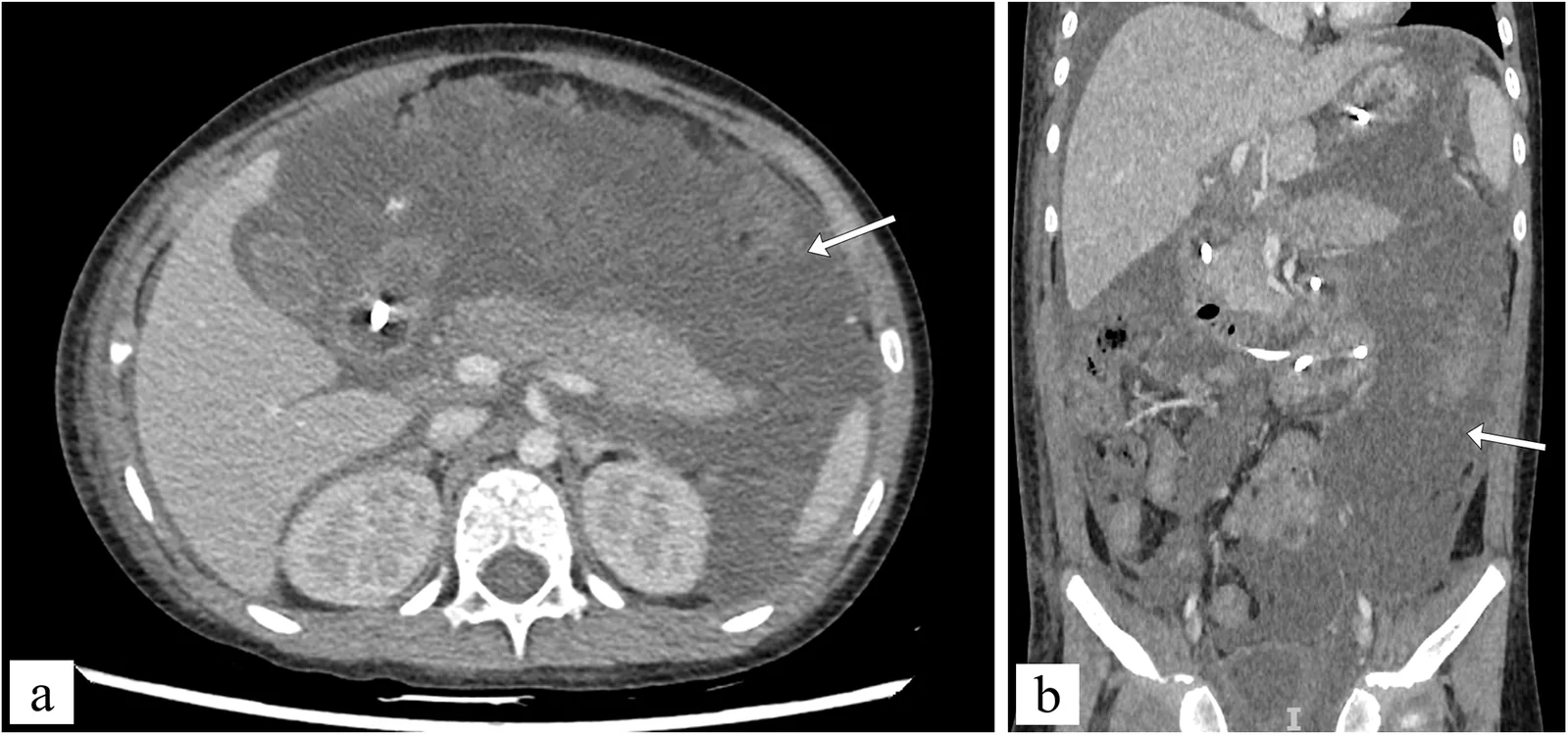
Abdominal CT at 1 week after onset. **(a)** Axial CT image; **(b)** Coronal CT image. Large hypodense areas of pancreatic and peripancreatic necrosis are visible, with necrotic fluid collections extending to the bilateral anterior pararenal spaces, left paracolic gutter, iliac fossae, and pelvic cavity (white arrow).

**Figure 2:**
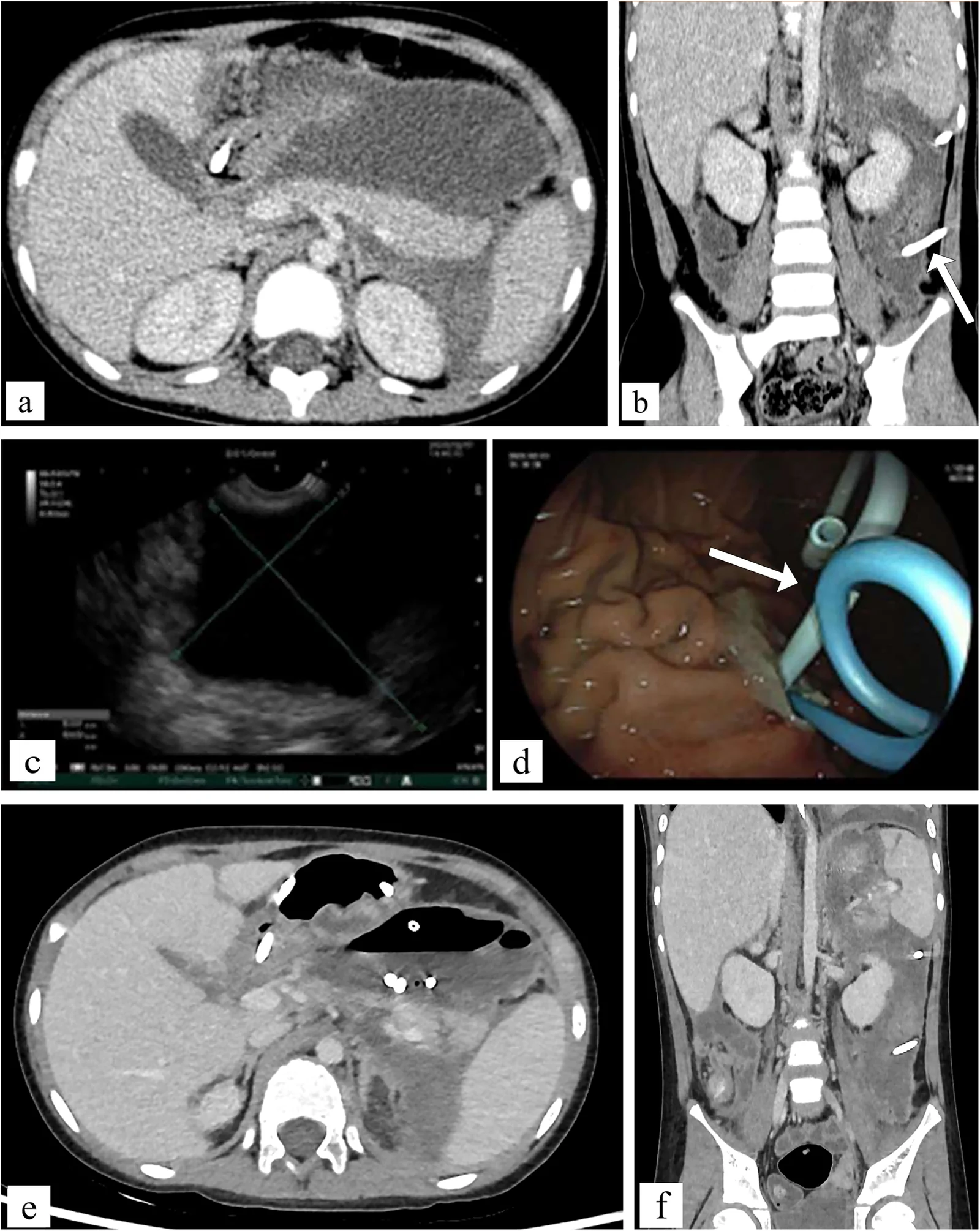
Stepwise management of extensive walled-off necrosis. **(a,b)** Axial and coronal CT images after CT-guided percutaneous drainage at 2−3 weeks post-onset. The drainage catheter is visible (white arrow). **(c,d)** Endoscopic ultrasonography-guided transmural drainage: EUS view of the necrotic cavity and placement of two double-pigtail plastic stents (white arrow). **(e,f)** Axial and coronal CT images following EUS-guided transmural drainage.

On day 40, ETN was performed due to persistent high fever, elevated inflammatory markers, and CT showing no reduction in collection size with necrotic tissue extensively distributed in the paracolic gutters and pelvic cavity. Before the procedure, enhanced CT was reviewed with radiologists to confirm the absence of significant vascular structures within the necrotic cavity. An adult-type therapeutic gastroscope (Olympus Q260J) was used. The endoscope was advanced through the gastric sinus tract into the lesser sac, where large amounts of pale yellow pus and necrotic tissue were observed. A 32 mm snare was used to retrieve necrotic tissue (Figures 3A–C). Since the pancreatic tail necrotic cavity communicated with the left paracolic gutter, the endoscope was navigated under direct visualization along the pancreatic tail direction into the left paracolic gutter. After adequate debridement of necrotic tissue, two percutaneous drainage catheters were seen penetrating the left peritoneum into the necrotic cavity (Figure 3D). Minor oozing from the wound surface was successfully controlled with hot biopsy forceps coagulation. Fluoroscopy and guidewire were not used, and no bleeding requiring intervention occurred. Each session lasted approximately 90 min, with approximately 80 cm^3^ of necrotic tissue removed in total across the two sessions. Pink granulation tissue served as the safety boundary. Two new double-pigtail plastic stents were reinserted after debridement.

**Figure 3:**
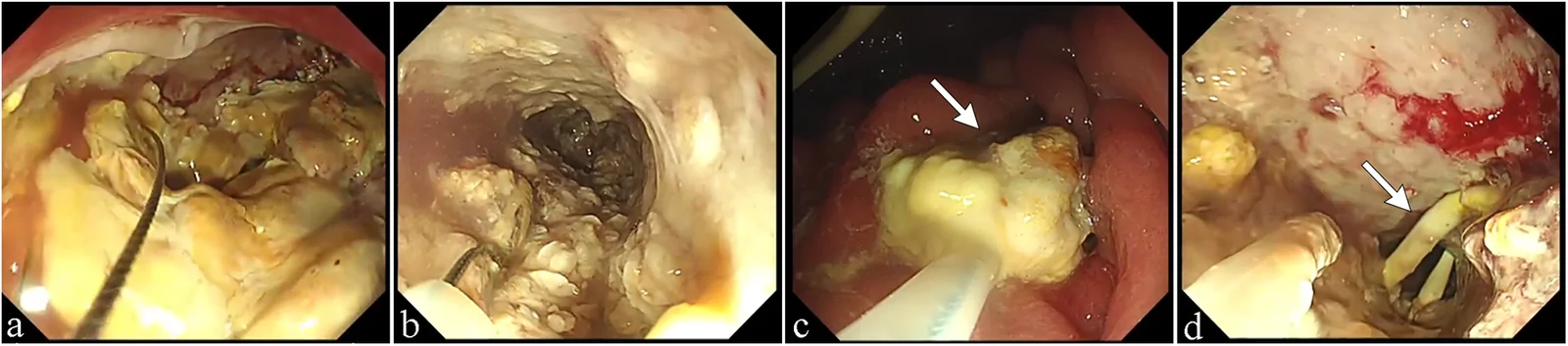
First session of endoscopic transgastric necrosectomy (ETN). **(a)** The endoscope enters the lesser sac via the stomach; **(b)** The endoscope is advanced to the left paracolic gutter; **(c)** A large amount of yellow necrotic tissue is retrieved using a snare (white arrow); **(d)** Two drainage catheters are seen penetrating the left peritoneum into the necrotic cavity of the paracolic gutter (white arrow).

After two sessions of ETN, the extensive necrotic tissue extending from the lesser sac to the paracolic gutter was successfully debrided, as confirmed by post-procedural CT (Figure 4). The two plastic stents were retrieved endoscopically three months later.

**Figure 4:**
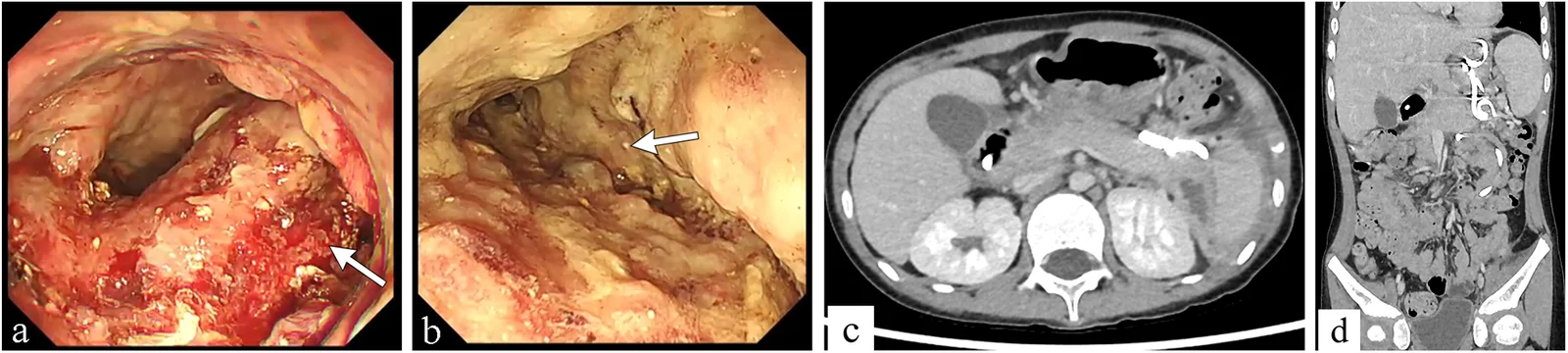
Follow-up after two sessions of ETN. **(a)** Endoscopic view of the lesser sac (white arrow); **(b)** Endoscopic view of the left paracolic gutter (white arrow); **(c)** Axial CT image; **(d)** Coronal CT image. These images confirm successful debridement of the extensive necrotic tissue extending from the lesser sac to the paracolic gutter.

## Discussion

Current management strategies for pancreatic collections in children closely parallel those employed in adults (11). Although endoscopic drainage has become a well-established modality for PFCs in the adult population, data regarding its efficacy and safety in pediatric patients remain limited (12). Active intervention is warranted when collections become infected. Surgical, percutaneous, and endoscopic approaches are all viable options, with the choice depending on the patient's clinical stability, the anatomical location of the collection, and the extent of necrosis (13, 14). For collections adjacent to the stomach or duodenum, EUS-guided drainage is recommended as the optimal approach. However, for multiloculated or extensive infected collections extending into cavities remote from the gastrointestinal lumen, a combination of endoscopic and percutaneous drainage is often a practical and necessary strategy (15). ETN has been increasingly recognized by the international surgical and gastroenterological community as a preferred first-line minimally invasive treatment for pancreatic necrosis; nevertheless, it is generally accepted that its indication is limited to necrotic tissue within the lesser sac, and that it is technically challenging to address necrosis extending to the paracolic gutters or pelvic cavity (16).

Percutaneous drainage is a safe treatment modality for WON. Independent predictors for requiring a step-up approach include a collection size greater than 10 cm, extension of peripancreatic collections to the paracolic gutter, and solid necrotic content exceeding 30% (17). The 2018 European Society of Gastrointestinal Endoscopy guidelines recommend that for patients with WON extending to the pelvic paracolic gutter, concurrent endoscopic transmural drainage and percutaneous drainage should be considered (8). A retrospective study by Baroud et al., which classified pancreatic necrotic collections based on the site of involvement, the amount of necrotic content, and the presence or absence of infection, demonstrated that patients with diffuse collections (extending to more than two sites) and/or a high percentage of solid debris (>30%) had a more complicated clinical course and prolonged hospital stay during ETN. Furthermore, in a retrospective series of 53 patients, larger WON, extension to the paracolic gutter, and pre-existing diabetes mellitus were associated with the need for surgical intervention (18).

In the present case, pancreatic and peripancreatic necrosis extended from the lesser sac to the paracolic gutter and iliac fossa. Although percutaneous drainage was performed two weeks after disease onset, the patient could not tolerate the serial dilation required for upsizing to a 30-Fr large-bore drainage catheter due to significant procedural pain. During ETN, we extended the conventional indication by leveraging the flexibility of the endoscope to navigate from the pancreatic tail necrotic cavity into the left paracolic gutter and pelvic cavity, thereby debriding necrotic tissue in these regions. This approach circumvented the need for additional combined percutaneous sinus endoscopy or video-assisted retroperitoneal debridement to manage necrosis in the paracolic gutter (19). These alternative procedures would have increased pain, hospitalization duration, and costs for the child.

In conclusion, this case highlights that extended ETN may be a safe and effective treatment option for extensive WON extending beyond the lesser sac in children. Further case accumulation and prospective studies are warranted to clarify the precise role of this approach in the management of extensive WON in the pediatric population.

## Data Availability

The original contributions presented in the study are included in the article/Supplementary Material, further inquiries can be directed to the corresponding author.
